# Personalized Radiation Therapy for Breast Cancer

**DOI:** 10.3390/curroncol31030121

**Published:** 2024-03-20

**Authors:** Waqar Haque, Edward Brian Butler, Bin S. Teh

**Affiliations:** Department of Radiation Oncology, Houston Methodist Hospital, Houston, TX 77030, USA; ebutler@houstonmethodist.org (E.B.B.); bteh@houstonmethodist.org (B.S.T.)

**Keywords:** breast cancer, radiation therapy, hypofractionated radiation, partial-breast radiation

## Abstract

Breast cancer is diagnosed in nearly 3 million people worldwide. Radiation therapy is an integral component of disease management for patients with breast cancer, and is used after breast-conserving surgery or a mastectomy to reduce the risk of a local recurrence. The following review describes the methods used to personalize radiation therapy by optimizing patient selection, using advanced treatment techniques to lessen the radiation dose to normal organs, and using hypofractionation in order to shorten the duration of radiation treatment.

## 1. Introduction

Breast cancer is the most common malignancy diagnosed in the world, with an estimated 2.6 million new patients being diagnosed in 2020 [1]. Postoperative radiation therapy (RT) is a mainstay of treatment for patients with breast cancer, with treatment typically being offered postoperatively following breast-conserving surgery (BCS) or, in select groups of patients, following a mastectomy [2]. The first study that demonstrated the safety and efficacy of postoperative RT following breast-conserving surgery to reduce the risk of a local recurrence was started in the UK in Guy’s Hospital, London in 1960, with subsequent trials investigating the use of postmastectomy radiation therapy starting in the US in 1971 [3,4,5,6]. These first trials in the UK delivered a dose of 38 Gy in 15 fractions to the target, whereas trials in the US used a dose of 50 Gy in 25 fractions, which was used in either the ipsilateral breast in patients undergoing breast-conserving surgery with a node-negative disease, or the chest wall and regional nodes including the axillary nodes, supraclavicular nodes, and internal mammary nodes in women undergoing mastectomy.

In the past 50 years, numerous advancements have been made with respect to the knowledge of tumor biology and its impact on outcomes for patients with breast cancer [7], radiation treatment delivery [8], and imaging capabilities both prior to radiation as well as with real-time imaging on the treatment machine [9]. These discoveries have enabled radiation oncologists to personalize RT for patients with breast cancer. The purpose of the present report is to provide a comprehensive overview of the personalization of RT for patients with breast cancer with respect to the optimal patient selection, RT technique, fractionation, and use of genomic biomarkers to potentially select for patients most likely to benefit from adjuvant radiation. This report also outlines future directions that aim to improve the therapeutic ratio and tailor RT to the exact needs of the patient.

## 2. Optimal Patient Selection

With the advent of improved screening mammographic techniques in the 1980s [10], breast cancers were detected at an earlier stage, leading investigators to question the need for RT in women with clinically occult breast cancers. This led to the NSABP B21 study in 1989, in which 1009 women with breast cancers measuring ≤ 1 cm receiving lumpectomy were randomized to be treated postoperatively with either RT, Tamoxifen and RT, or observation [11]. The results showed a statistically significant increase in local control with the use of RT, which led the investigators to conclude that RT could not be omitted in women with small (≤1 cm) breast cancers. The next major breakthrough with respect to optimizing patient selection amongst patients with early-stage breast cancer came from the CALGB 9343 trial [12]. In this study, 636 women with T1N0 breast cancer, an ER+ disease, and aged 70 or greater were treated with lumpectomy and then randomized to receive either RT and Tamoxifen or Tamoxifen alone. After 10 years, the cohort of patients treated with RT had a 98% local control (LC) rate, compared to a 90% LC rate in patients not receiving RT, though no difference in overall survival (OS) was noted. This trial laid the foundation for allowing the omission of radiation therapy in women aged ≥ 70 with an ER+ disease following breast-conserving surgery, despite demonstrating improved LC rates with the use of radiation.

Similar conclusions were reached in the PRIME 2 trial [13], in which 1326 women with ER+ breast cancer, a T1-2N0 disease (but ≤3 cm), and aged ≤ 65 years were treated with breast-conserving surgery and then randomized to receive RT or no RT. All women received endocrine therapy. The 10-year failure rate was 10% in the no-RT group, compared to 1% in the RT group, and again no difference in OS was observed. A third study, the LUMINA trial, sought to incorporate the disease’s luminal status in order to select for patients in whom the omission of RT may be reasonable following breast-conserving surgery [14]. In this single-arm prospective trial, 501 women aged ≥ 55 years who were treated with breast-conserving surgery with a Grade 1–2 T1-2N0 and luminal A-subtype (defined as ER+PER+HER2− and Ki67 ≤ 13.25%) disease were treated with adjuvant endocrine therapy with no postoperative radiation. At a 5-year follow-up, the recurrence rate was 2.3%, which led the investigators to conclude that this patient population could be considered for the omission of RT. However, caution should be undertaken before routinely omitting radiation treatment following breast-conserving surgery in women as young as 55 years of age, since the LUMINA trial was only a single-arm trial and had a limited follow-up time. Of note, to date there has not been a randomized Phase III study that has demonstrated that the use of postoperative RT does not improve the LC rate; however, in certain subsets of patients, primarily in patients aged ≥ 70 with an ER+ disease, the current NCCN guidelines do allow for omission based on the CALGB 9343 data. Currently, nearly 50% of women with T1N0 ER+ breast cancer do not receive adjuvant RT following breast-conserving surgery [15]. Furthermore, one criticism of these trials comparing adjuvant radiation and endocrine therapy to endocrine therapy alone is they do not account for patient noncompliance with endocrine therapy. With the compliance rate of adjuvant endocrine therapy reported to be as low as 40% [16], it is possible that women who are prescribed endocrine therapy in real-world settings may not be fully receiving the prescribed treatment, leading to potentially higher risks of recurrence. One meta-analysis has demonstrated similar local control rates and breast-cancer-specific survival rates with the use of adjuvant hormonal therapy compared to the use of adjuvant radiation therapy [17]. A current gap in the literature is the lack of data comparing radiation alone to endocrine therapy alone as adjuvant treatments for patients with early-stage breast cancer.

## 3. Radiation Therapy Technique

**Figure 1:**
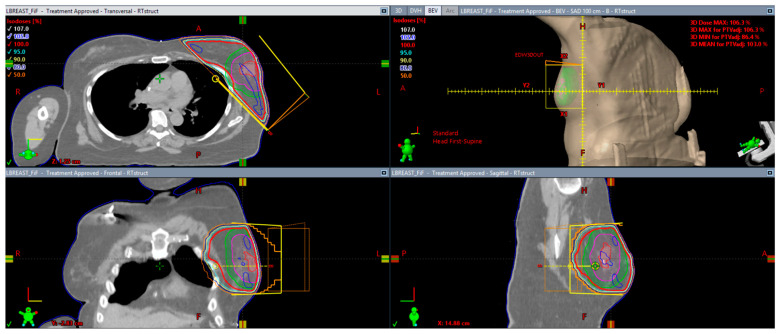
Partial-breast radiation using tangents to cover only the tumor bed with margins. The red line that encompasses the breast and the blue lines and the medial and lateral points within the breast are isodose lines that represent the relative dose deposited in the partial breast.

RT to the breast was initially delivered with the use of two-dimensional tangent beams, including a medial beam and a lateral beam. Advances in RT delivery and image guidance have allowed radiation oncologists to use advanced radiation techniques to deliver more conformal treatment, and in some cases, treat only the tumor bed as opposed to the entire breast, which can lead to decreased doses to the surrounding normal structures. Recurrence patterns showing that most ipsilateral breast tumor recurrences occurred in the region of the breast led to investigators studying the use of partial-breast radiation, initially delivered over the course of one week [18]. Partial-breast radiation could be delivered using either brachytherapy and interstitial catheters or by using an implantable balloon device, in which case the dose delivered was 34 Gy in 10 bid/twice-daily fractions delivered over the course of one week [19], or external beam radiation therapy, typically to a dose of 38.5 Gy in 10 bid/twice-daily fractions, also delivered over the course of one week [20] (Figure 1).

The different fractionation schemes used to administer partial-breast radiation include 30 Gy over 5 fractions given every other day over the course of two weeks, used by investigators in Florence, Italy [21], or 40 Gy delivered over the course of 15 fractions given once per day over the course of three weeks, as demonstrated by investigators from the UK in the Import Low trial [22].

The NSABP B-39 trial was a randomized trial that sought to compare whole-breast radiation to accelerated partial-breast irradiation (APBI) in women with breast cancer following breast-conserving surgery. The entry criteria for this trial were broad, and included patients with a Stage 0–II disease. Patients with a node-positive disease were allowed to be included, though only up to three nodes could be positive. At a median follow-up of 10.2 years, the incidence of local failure in the group of patients that received APBI was 4.6%, compared to 3.9% in the whole-breast radiation cohort. This did not meet the prespecified criteria for equivalence [23]. However, as the local failure rate at 10 years differed by <1%, the authors did conclude that APBI was a reasonable option for some patients. The inclusion criteria for this trial were likely too broad and included patients who would be expected to benefit from whole-breast radiation, including patients with node-positive and triple-negative diseases. Optimal patient selection is critical to ensure that treatment of the partial breast does not jeopardize local control for these patients, and it is likely that APBI is safe and effective in patients with a T1N0 ER+ disease. To this extent, the American Society of Radiation Oncology has published guidelines regarding patient characteristics for the suitability of APBI [24]. Specifically, patients who are suitable candidates for APBI include those aged ≥ 50 years, those with surgical margins negative by at least 2 mm, and those with a Tis or T1N0 disease, with DCIS only being deemed as suitable if it is of a low to intermediate grade with a size of ≤2.5 cm with surgical margins of at least ≥ 3 mm.

Additional advanced radiation techniques deliver radiation to the whole breast while minimizing the dose to the surrounding normal structures. This is especially important as excess radiation doses delivered to the heart can lead to an increased risk of adverse cardiac events [25]. One of the advanced techniques used to personalize RT and to limit the dose to the heart is deep inspiration breath hold (DIBH) [26]. In this radiation technique, RT is delivered during maximal inspiration, which causes the heart to shift inferiorly while the chest wall moves anteriorly. This increased distance between the heart and the chest wall can result in a significantly reduced dose deposited in the heart [27]. An additional radiation technique that can be used to reduce the cardiac dose is prone breast radiation [28], in which patients lay in the prone position during their daily radiation treatment. This allows the breast to be displaced away from the heart, which can lead to a reduction in the radiation dose administered to the heart, lung, and other internal organs. Intensity-modulated radiation therapy (IMRT) is a third option that can be utilized for select patients to reduce cardiac toxicity [29]. IMRT utilizes inverse planning algorithms that can lead to radiation beams delivering doses conformally around concave structures, which can decrease the dose to the heart particularly in cases when treatment of a more extensive volume including the internal mammary nodes is required [29]. Practically, this would be expected to decrease the incidence of cardiac events, and techniques to spare the heart have been demonstrated to have both dosimetric and clinical benefits in decreasing cardiac dysfunction following radiation therapy [30]. Proton-based radiation therapy is another technique that may be able to better spare internal organs such as the heart, and in doing so, it may reduce the long-term toxicity associated with radiation therapy [31]. However, no randomized trials comparing proton-based radiation to photon-based radiation for the treatment of breast cancer have been completed, and the Particle Therapy Cooperative Group has concluded that no studies have demonstrated that proton-based radiation improves outcomes compared to photon-based radiation [32].

## 4. Fractionation

The first breast cancer RT trials in the UK treated patients to a dose of 38 Gy in 15 fractions, whereas the trials in the US utilized a dose of 45–50 Gy in 1.8–2 Gy daily fractions with the option of a boost that was delivered over the course of 1 week [3,4,5], for a total of 5–6 weeks of radiation therapy. The length of radiation treatment can potentially lead to logistical challenges and increased costs for patients, which has led investigators to determine if hypofractionated radiation therapy could be utilized without compromising the safety and efficacy of this treatment. The aforementioned APBI trials did demonstrate that an accelerated course of radiation could be delivered over the course of 1 week without compromising safety, but this was only deemed to be a suitable option in a subset of early-stage patients [24].

The radiation sensitivity of different types of tissue can be described by the alpha–beta ratio, in which a lower alpha–beta ratio suggests tissue that has a slower proliferation rate [31]. While certain cancers, such as oropharyngeal cancers, have high alpha–beta ratios and are beneficiaries of treatment with smaller fraction sizes, tumors such as those in breast cancer typically have a low alpha–beta ratio of 4, and consequently can have an improved therapeutic ratio with the use of hypofractionated radiation therapy [33,34]. The first randomized trials to demonstrate the safety and efficacy of hypofractionated whole-breast radiation were published by Canadian investigators in 2002 [35]. These researchers randomized 1234 women with T1-2N0 breast cancer following breast-conserving surgery to receive a radiation therapy dose of either 50 Gy in 25 fractions or 42.5 Gy in 16 fractions. Their results demonstrated no differences in the local control rates or toxicity rates, leading the investigators to conclude that the shorter course was a reasonable option for patients. The demonstration of medical equipoise between conventionally fractionated radiation and hypofractionated radiation has been a paradigm shift for the management of breast cancer. By demonstrating that RT could be delivered for a shorter duration without compromising the clinical outcome ushered in the era of hypofractionation, leading investigators to further explore even shorter fractionation schedules.

The UK Start A trial randomized 2236 women with T1-T3N0-N1 breast cancer treated with either lumpectomy or mastectomy to receive a postoperative radiation dose of either 50 Gy in 25 fractions, or 41.6 Gy in 13 fractions, or 39 Gy in 13 fractions [36]. There were no differences in the local control between either of the treatment arms. Cosmetic appearances appeared no different between the 50 Gy and 41.6 Gy arms, though they were rated worse in the 50 Gy arm when compared to the 39 Gy arm. The UK Start B trial randomized 2215 women with T1-3 N0-1 breast cancer who were treated with either breast-conserving surgery or mastectomy to receive adjuvant radiation to a dose of either 50 Gy in 25 fractions or 40 Gy in 15 fractions [37]. No difference in the local control was observed between the two groups, and fewer cosmetic toxicities were observed in the 40 Gy group. In the UK Fast trial, investigators sought to decrease the number fractions from 15 to 5. A total of 915 women with T1-2N0 breast cancer following lumpectomy were randomized to receive postoperative radiation to a dose of either 50 Gy in 25 fractions over the course of 5 weeks or 30 or 28.5 Gy in 5 fractions given once per week [38]. No significant differences were observed in the local control between the three patient groups. Cosmetic appearances appeared similar between the group of patients treated with 50 Gy in 25 fractions and those treated with 28.5 Gy in 5 fractions, though worse cosmetic toxicity was observed in the 30 Gy cohort. In the UK Fast Forward trial, 4096 women with T1-3N0-1 breast cancer treated with either lumpectomy or mastectomy were randomized to receive adjuvant radiation to a dose of either 40 Gy in 15 fractions over 3 weeks, 27 Gy in 5 fractions over 1 week, or 26 Gy in 5 fractions over 1 week [39]. After five years, there were no differences in local control observed between the three patient cohorts, and no difference in the cosmetic outcomes was observed between the 40 Gy and 26 Gy cohorts. However, a worse cosmetic outcome was observed in the group of patients treated with 27 Gy in five fractions. The use of hypofractionation has been demonstrated to have minimal toxicity, with modern series demonstrating no Grade 3 toxicities and a <10% incidence of edema or telangiectasia [40].

The aforementioned hypofractionation trials were conducted primarily in patients treated with breast-conserving surgery. The UK Start A and B trials and the UK Fast Forward trial did allow the inclusion of patients who had been treated with mastectomy, but only <15% of patients in each trial had received a mastectomy. One trial conducted in China investigated the safety and efficacy of hypofractionated radiation specifically amongst patients treated with mastectomy [41]. In this trial, 820 patients with T3-4N2-3 breast cancer were treated with mastectomy and randomized to chest wall and nodal irradiation to a dose of either 50 Gy in 25 fractions or 43.5 Gy in 15 fractions. There were no differences in the local control or acute or late toxicity, leading the investigators to conclude that hypofractionation is safe for patients undergoing mastectomy. A smaller multi-institutional trial from America attempted to determine the safety and efficacy of hypofractionation in women undergoing mastectomy [42]. A total of 69 women with Stage II–IIIA breast cancer treated with mastectomy were treated to a dose of 36.6 Gy in 11 fractions with the option of a 4- fraction boost. Grade 2 or higher late toxicities were 12%, and the local failure rate after a median follow-up of 54 months was 4.6%, leading the authors to conclude that this may be a fractionation scheme worthy of further study.

The current NCCN guidelines recommend hypofractionation, defined as 40–42.6 Gy in 15–16 fractions, for use in patients receiving whole-breast radiation only following breast-conserving surgery, with ultra-hypofractionation, defined as 28.5 Gy in 1 fraction per week, reserved for use in patients aged 50 or greater with an early-stage, node-negative disease [2]. While five-fraction whole-breast radiation delivered in one week, as per the UK Fast Forward trial, is not yet an acceptable fractionation scheme per the NCCN guidelines, it is expected that this may be allowed once 10-year follow-up data are available. Conventionally fractionated radiation to a dose of 45–50 Gy in 1.8–2 Gy per fraction is recommended in patients receiving adjuvant radiation to the regional nodes in addition to the breast or in patients receiving postmastectomy RT, while hypofractionation in 15–16 fractions is allowed in patients not undergoing reconstruction.

## 5. Genomic Personalization of Radiation Therapy

Investigators have sought to discover multigene expression assays that can predict for local recurrence after breast-conserving surgery. The ability to select for the patients most likely to experience a recurrence following breast-conserving surgery may optimize treatment by allowing for the de-escalation and omission of RT in women unlikely to experience recurrence, and allowing for treatment escalation in the patients found to have a higher risk of local recurrence. The 21-gene Oncotype recurrence score (RS) is a genomic test that predicts for the distant metastasis rate at 10 years with the use of endocrine therapy for patients with ER+HER2- breast cancer. This can predict for the absolute benefit of chemotherapy and allows physicians to select for patients who are most likely to benefit from chemotherapy [43]. The quest to identify such a genomic test that can select for patients most likely to benefit from RT is ongoing.

The first genomic assay to predict for locoregional recurrence was the RS. In 2010, using data from 895 node-negative patients with breast cancer treated on NSABP-B14 and NSABP-B20 treated with either Tamoxifen, a placebo, or Tamoxifen and the placebo, Mamounas et al. explored the association between RS and local recurrence, and demonstrated that the risk of recurrence was 4% in patients with a low RS, 7.2% in patients with an intermediate RS, and 15.8% in patients with a high RS [44]. Subsequently, Mamounas et al. sought to determine whether the RS was also associated with local recurrence in a node-positive breast cancer patient population [45]. The investigators retrospectively reviewed outcomes for 1065 ER+ Tamoxifen-treated patients with node-positive breast cancer and showed that the RS was associated with local recurrence risk, with local recurrence rates of 3% in the low-RS group, 7.2% in the intermediate-risk group, and 12.2% in the high-risk group. Population-based studies have since shown that the RS can be used to select for patients most likely to benefit from postoperative RT out of all patients [46], and importantly also in women aged ≥ 70 [47]. The RS is a surrogate for aggressive tumor biology and it is likely that prospective data will validate the predictive ability of the RS to select for patients unlikely to benefit from postoperative RT following breast-conserving surgery.

An additional genomic signature that has been shown to predict for local recurrence following breast-conserving surgery is PAM-50 [48]. This is a genomic test based on 50 cancer genes that uses RT-PCR to assign a recurrence score for patients. Australian investigators obtained tumor blocks from 1204 patients enrolled in the ABCSG 8 randomized trial. Of note, 1034 of these patients received RT. Patients who had a low risk of recurrence (ROR) according the PAM-50 analysis were found to have a 0.9% risk of recurrence after 10 years, compared to a 3.8% risk of recurrence after 10 years in patients with a high ROR in the PAM-50 analysis. However, this test was not found to be predictive for the benefit of RT. A promising genomic signature that may predict for patients with ER+HER2- breast cancer with favorable tumor biology in whom radiation therapy may be able to be omitted is the 16-gene Profile for the Omission of Local Adjuvant Radiation (POLAR) [49]. Investigators first trained the POLAR genomic signature in 243 patients from the SweBCG91-RT cohort, and then validated the test in 354 patients from this same group of patients. Patients with low-risk POLAR scores were found to have a 10-year local recurrence rate of 6%, and no significant benefit was observed with the use of RT, leading the investigators to conclude that the POLAR test may select candidates suitable for the omission of radiation therapy. The POLAR test has not yet been validated using randomized data, and is not ready for routine clinical use, but ongoing randomized trials will demonstrate its predictive ability.

Other researchers have sought to discover genes that may predict for radiation sensitivity. A radiosensitivity predictive assay was first developed in 2009 for head-and-neck cancer, rectal cancer, and esophageal cancer [50]. Subsequently, investigators at the University of Michigan used clonogenic survival assays to generate a radiation sensitivity signature (RSS) that identified genes associated with radioresistance [51]. The authors of the study concluded that the RSS could be used to select for patients with tumors refractory to standard radiation and therefore personalize radiation by potentially selecting for patients who would benefit from treatment escalation. Researchers from Stanford created an intrinsic radiosensitivity gene signature that would stratify patients into either radiation-sensitive or radiation-resistant subgroups [52]. These investigators demonstrated in a validation cohort that patients in the radiation-sensitive subgroup had improved local control following the use of radiation therapy, while no benefit with the use of radiation therapy was observed in the radiation-resistant subgroup. While these studies have retrospectively demonstrated that radiation sensitivity signatures may predict for patients most likely to benefit from radiation therapy, these genomic studies have not yet been prospectively validated and are not yet used clinically for risk stratification of patients.

## 6. Future Directions

The aforementioned studies have demonstrated the potential of genomic assays to personalize radiation therapy amongst patients with breast cancer. Several ongoing studies are attempting to prospectively validate the predictive utility of genomic assays. Table 1 lists trials seeking to determine patient populations in which the omission of radiation therapy may be safe following breast-conserving surgery.

One of these is a single-arm prospective trial sponsored by the University of Michigan and other US institutions called the “Individualized Decisions for Endocrine Therapy Alone” (IDEA) trial [53]. Somewhat similar to the Lumina trial, in this study, postmenopausal woman aged 50–69 with T1N0 ER+HER2- breast cancer treated with breast-conserving surgery and an RS ≤ 18 will receive adjuvant endocrine therapy alone and radiation therapy will be omitted. A second observational trial is the “Profiling early breast cancer for radiotherapy omission” (PRECISION) trial [54]. In this Phase II trial conducted by the Dana–Farber Cancer Institute, women aged 50–75 with T1N0, ER-HER2- breast cancer treated with breast-conserving surgery and receiving endocrine therapy with a low PAM-50 score are given the option to omit radiation. If the women opt for omission, they are enrolled in the study and observed with the goal of determining the 5-year local recurrence rate. An additional prospective study, the “Post-operative avoidance of radiotherapy: biomarker selection of women categorized to being a very low-risk group by IHC4+C” (PRIMETIME) study [55], is being sponsored by the Institute of Cancer Research of the United Kingdom with a goal of enrolling 1550 women. Women eligible for enrollment are those aged 60 or over with T1N0 breast cancer, an ER+HER2- disease, and who have been treated with breast-conserving surgery. Their IHC4+C score is determined using ER, PR, HER2, and the Ki-67 score to determine the risk of distant recurrence, and the patients are stratified into very-low-risk, low-risk, intermediate-risk, and high-risk groups. Women with a low-risk score will be observed, while women in the other subgroups will receive adjuvant radiation therapy. The primary endpoint of this study is 5-year local recurrence.

Two large Phase IIII randomized trials are currently enrolling patients to determine whether genomic tests can be used to select for patients with breast cancer in whom postoperative radiation can be safely omitted. The “DE-escalation of Breast Radiation” (DEBRA, NRG-BR007) study [56] is a multicenter Phase III study sponsored by NRG Oncology that is seeking to enroll 1670 women with T1N0 breast cancer with an ER+HER2- disease and an RS score ≤ 18. Women meeting these criteria are randomized to receive either endocrine therapy alone or endocrine therapy and radiation therapy, and will then be followed for 10 years, with the goal of determining whether the omission of radiation therapy is non-inferior to treatment with postoperative radiation. The “Examining Personalized Radiation Therapy for low-risk early breast cancer” (EXPERT) [57] trial is being conducted in Australia and New Zealand with a goal of enrolling 1170 women. In this Phase III non-inferiority trial, women with T1N0 breast cancer and ER+PR+HER2- breast cancer with a Grade 1–2 disease treated with breast-conserving surgery and a low-risk PAM-50 score are randomized to receive either endocrine therapy alone or radiation therapy and endocrine therapy.

Postmastectomy radiation therapy (PMRT) is currently recommended for women with a node-positive disease and is also considered for patients with T3N0 breast cancer [2]. This is based on postmastectomy radiation therapy trials that showed benefits in both overall survival and local control with the inclusion of PMRT in this patient population [58,59]. However, retrospective data have demonstrated that the RS can predict for local recurrence in patients with a node-positive disease, which suggests that the RS could possibly predict for patients in whom PMRT, or regional node irradiation following breast-conserving surgery, could be omitted [44]. This has led to the MA 39 TAILOR RT trial, which is a multi-institutional Phase III trial randomizing patients with a low-risk node-positive T3N0 disease to receive either regional nodal radiation or no regional nodal irradiation [60]. In this trial, patients undergoing breast-conserving surgery will be randomized to receive treatment with either breast-only radiation or radiation to the breast and regional nodes. Patients receiving mastectomy treatment will be randomized to receive either PMRT or no PMRT.

Neoadjuvant chemotherapy is used to downsize tumors prior to surgery and can enable a greater proportion of women to be candidates for breast-conserving surgery [61]. Though there is high level of data that support the use of PMRT in patients with a node-positive disease [57], the data supporting PMRT in this patient population were largely obtained prior to the era of neoadjuvant chemotherapy. Whether PMRT is beneficial in patients with a clinically node-positive disease who have a pathologic complete response in their lymph nodes following neoadjuvant chemotherapy is currently not known, though guidelines support the treatment of these patients based on their clinical nodal stage [2]. A retrospective review from the National Cancer Database demonstrated that in patients with a node-positive disease were found to have a pathologic complete response in their lymph nodes following neoadjuvant chemotherapy, but there was no difference in overall survival following PMRT noted in patients with a cN1 disease [62]. However, patients with a cN2 or cN3 disease who converted to ypN0 after neoadjuvant chemotherapy were found to have improved overall survival following PMRT. The NSABP B51 trial is a Phase III randomized trial that enrolled patients with T1-3N1 breast cancer treated with neoadjuvant chemotherapy who had a pathologic complete response in their lymph nodes [63]. If these patients underwent a mastectomy, they were randomized to receive either chest wall and nodal radiation or no radiation, and if these patients received breast-conserving surgery, they were randomized to receive either adjuvant breast and regional nodal radiation or postoperative radiation only to the breast. The trial is now closed and is not accruing further participants, though its results have not yet been reported. A second trial, NRG BR008, or the HERO trial, is a multi-institutional Phase III trial that is seeking to optimize the use of postoperative radiation in women with HER2+ breast cancer following breast-conserving surgery [64]. Women with T1-2 (≤3 cm) N0 breast cancer with an HER2+ disease undergoing neoadjuvant HER2-based targeted therapy who have a pathologic complete response after breast-conserving surgery will be randomized to receive either postoperative radiation or no radiation.

Hypofractionated radiation therapy is currently the standard of care for postoperative radiation after breast-conserving surgery for patients receiving RT to the breast. The previously mentioned UK Start A and B trials, and the Fast Forward trial [38,39], did include patients who underwent mastectomies, but this represented only 8–12% of the treated patients. The study by Wang et al. did demonstrate the safety and efficacy of hypofractioned postmastectomy radiation therapy [35], but whether hypofractionated RT can be safely administered in patients undergoing mastectomy and reconstruction treatment is an active research question. The ALLIANCE A221505 RT CHARM trial is a Phase III randomized trial that enrolled 800 patients with breast cancer who were treated with mastectomy reconstruction and postoperative radiation to the chest wall and regional nodes to a dose of either 50 Gy in 25 fractions or 42.56 Gy in 16 fractions [65]. The primary objective of this trial is to determine whether the reconstruction complication rate at 24 months post RT is non-inferior with hypofractionation.

Despite the advances that have been made in optimizing the personalization of radiation therapy for patients with breast cancer, there are several unanswered questions that current investigators are hoping to answer. The aforementioned studies will help to determine if there is a genomic test that can predict for patients who may be able to safely omit radiation from their treatment following breast-conserving surgery, if PMRT is required following breast-conserving surgery, if regional nodal radiation is required in cases of low-risk node-positive breast cancer, and if hypofractionated radiation therapy can safely be used in patients receiving mastectomy and breast reconstruction.

## 7. Conclusions

Postoperative RT has been utilized to reduce the risk of local recurrence for women with breast cancer for over 50 years. Initially, RT was recommended for all women receiving breast-conserving surgery, with treatment encompassing the entire breast and given over the course of 5–6 weeks. Decades of research has allowed physicians to personalize radiation therapy by demonstrating the safety of omitting radiation therapy for certain subgroups of patients and the efficacy of partial-breast radiation as well as hypofractionation. In the future, it is expected that genomic assays will be used to further personalize RT and improve the therapeutic ratio by selecting for patients who will likely derive the most benefit from the use of radiation.

## Figures and Tables

**Table 1:** Trials exploring the omission of radiation.

	IDEA	PRECISION	PRIMETIME	DEBRA/NRG BR007	EXPERT	HERO/NRG BR 008
Target patient accrual	202	672	2400	1670	1167	1300
Estimated date of completion	2026	2026	2027	2026	2024	2034
Age of patients	50–69	50–75	≥60	50–70	≥50	≥40
Inclusion	ER+HER2-, RS ≤ 18, T1N0	ER+HER2-, T1N0, PAM 50	ER+HER2-, T1N0, IHC4+C	ER+HER2-, T1N0, RS ≤ 18	ER+HER2-, T1N0, PAM 50	HER2+, T1N0 or (if neoadjuvant) T2N0 (<3 cm), HER2-directed therapy
Treatment arms	Single-arm, prospective, omission	Omission if low PAM 50; radiation if intermediate or high PAM 50	Omission if very low IHC4+C; radiation if low, intermediate, or high IHC4+C	Observation or radiation	Observation or radiation	Observation or radiation
